# Generation of mesenchymal stromal cells from cord blood: evaluation of in vitro quality parameters prior to clinical use

**DOI:** 10.1186/s13287-016-0465-2

**Published:** 2017-01-24

**Authors:** Eliana Amati, Sabrina Sella, Omar Perbellini, Alberta Alghisi, Martina Bernardi, Katia Chieregato, Chiara Lievore, Denise Peserico, Manuela Rigno, Anna Zilio, Marco Ruggeri, Francesco Rodeghiero, Giuseppe Astori

**Affiliations:** 1 Advanced Cellular Therapy Laboratory – Hematology Unit, S. Bortolo Hospital – ULSS 6, Contra’ San Francesco 41, 36100 Vicenza, Italy; 2 Transfusion Medicine, S. Bortolo Hospital, Vicenza, Italy; 3 Hematology Project Foundation, Vicenza, Italy; 4 Genetics and Molecular Biology, Transfusion Medicine, S. Bortolo Hospital, Vicenza, Italy

## Abstract

**Background:**

Increasing evidence suggests the safety and efficacy of mesenchymal stromal cells (MSC) as advanced therapy medicinal products because of their immunomodulatory properties and supportive role in hematopoiesis. Although bone marrow remains the most common source for obtaining off-the-shelf MSC, cord blood (CB) represents an alternative source, which can be collected noninvasively and without major ethical concerns. However, the low estimated frequency and inconsistency of successful isolation represent open challenges for the use of CB-derived MSC in clinical trials. This study explores whether CB may represent a suitable source of MSC for clinical use and analyzes several in vitro parameters useful to better define the quality of CB-derived MSC prior to clinical application.

**Methods:**

CB units (*n* = 50) selected according to quality criteria (CB volume ≥ 20 ml, time from collection ≤ 24 h) were cultured using a standardized procedure for CB-MSC generation. MSC were analyzed for their growth potential and secondary colony-forming capacity. Immunophenotype and multilineage differentiation potential of culture-expanded CB-MSC were assessed to verify MSC identity. The immunomodulatory activity at resting conditions and after inflammatory priming (IFN-γ-1b and TNF-α for 48 hours) was explored to assess the in vitro potency of CB-MSC prior to clinical application. Molecular karyotyping was used to assess the genetic stability after prolonged MSC expansion.

**Results:**

We were able to isolate MSC colonies from 44% of the processed units. Our results do not support a role of CB volume in determining the outcome of the cultures, in terms of both isolation and proliferative capacity of CB-MSC. Particularly, we have confirmed the existence of two different CB-MSC populations named short- and long-living (SL- and LL-) CBMSC, clearly diverging in their growth capacity and secondary colony-forming efficiency. Only LL-CBMSC were able to expand consistently and to survive for longer periods in vitro, while preserving genetic stability. Therefore, they may represent interesting candidates for therapeutic applications. We have also observed that LL-CBMSC were not equally immunosuppressive, particularly after inflammatory priming and despite upregulating priming-inducible markers.

**Conclusions:**

This work supports the use of CB as a potential MSC source for clinical applications, remaining more readily available compared to conventional sources. We have provided evidence that not all LL-CBMSC are equally immunosuppressive in an inflammatory environment, suggesting the need to include the assessment of potency among the release criteria for each CB-MSC batch intended for clinical use, at least for the treatment of immune disorders as GvHD.

**Electronic supplementary material:**

The online version of this article (doi:10.1186/s13287-016-0465-2) contains supplementary material, which is available to authorized users.

## Background

Mesenchymal stromal cells (MSC) comprise a heterogeneous population of multipotent progenitor cells used in clinic for their immunomodulatory properties and their supportive role in hematopoiesis. Three main criteria have been proposed by the International Society for Cellular Therapy (ISCT) for MSC definition: (1) adherence to plastic under standard culture conditions; (2) expression of CD105, CD73, CD90, and lack of expression of HLA-DR, together with the hematopoietic and endothelial surface markers CD14, CD45, CD34, CD11b, and CD31; (3) in vitro differentiation potential into osteocytes, chondrocytes, and adipocytes under appropriate culture conditions [1].

MSC are potent modulators of immune responses, by virtue of direct cell-cell contact and production of poorly defined soluble factors [2–4]. MSC are not constitutively inhibitory, but acquire their immunosuppressive functions following priming by inflammatory cytokines, mainly interferon gamma (IFN-γ) and tumor necrosis factor alpha (TNF-α) [5, 6]. The inducible MSC immunoregulatory properties are shared by MSC from bone marrow (BM) and other tissues, as well as by more differentiated fibroblasts [7].

The amenability to ex vivo expansion and the immunomodulatory activity of MSC have encouraged extensive studies paving the way for their therapeutic use, in the context of hematopoietic stem cell transplantation (HSCT) and other clinical settings [8–10]. Since 2004, the use of cryopreserved allogeneic MSC for the treatment of steroid-refractory acute graft-versus-host disease (aGvHD) has become medical practice in many countries [11, 12].

Although BM remains the most common source, MSC can be isolated from various human tissues [13–15]. Particularly, cord blood (CB) represents an alternative source, which can be collected noninvasively and without major clinical concerns. The network of public CB banks worldwide provides an easy-to-access system for the use of fresh CB units for MSC generation when they are not suitable for banking, so that CB-derived MSC can be expanded and cryopreserved in advance with enormous clinical advantages.

CB-MSC display peculiar morphological, differentiative and trophic properties [16, 17]. Some authors demonstrated a higher proliferative potential of CB-MSC compared with BM- or adipose tissue-derived MSC, together with a normal karyotype after prolonged expansion [18–20]. More recently, the existence of distinct stromal CB populations with different performances in vitro has been postulated, on the basis of their proliferative potential, colony-forming efficiency, and telomere length [21]. Fewer studies have comprehensively addressed the immunomodulatory properties of CB-MSC, exerted on several T cell subsets and NK cells, but also through inhibition of dendritic cell function [20, 22–25].

To date, the low estimated frequency and the inconsistency of successful isolation are open challenges for the use of CB-MSC in clinical trials [26–28]. Most authors over the last years have suggested that CB volume and time from collection should be considered for a successful CB-MSC isolation [20, 29–31]. Recent studies have proposed efficient methods to obtain CB-MSC, avoiding strict quality selection of the starting material. These methods combined the traditional MNC separation or CB immunodepletion with the addition of variable supplements or coating strategies to support MSC growth [32, 33]. In this regard, the use of dexamethasone at the beginning of the culture has proven to inhibit monocyte adhesion and support CB-MSC proliferation [20, 33, 34], without inducing changes in the subsequent differentiation potential [35].

The present study aimed at obtaining MSC from CB, by means of an isolation procedure based on the transient use of dexamethasone as medium supplement. An essential goal was to analyze several in vitro parameters useful to define the quality of CB-derived MSC in view of their clinical use. Ultimately, the immunomodulatory function during the inflammation process was assessed as a measure of their in vitro potency, with the aim to improve cell characterization.

## Methods

### Cord blood collection

CB was collected after maternal informed consent from the Department of Transfusion Medicine, San Bortolo Hospital (Vicenza, Italy). CB units were collected from full-term deliveries by venipuncture immediately after cord clamping and before the delivery of placenta (in utero), then stored in bags containing 30 ml of citrate phosphate dextrose (Fresenius-Kabi, Bad Homburg vor der Höhe, Germany). Only CB units not suitable for banking with a net volume higher than 20 ml were processed within 24 hours from the collection. Clinical information from each donor including pregnancy details and CB parameters was prospectively collected.

### CB-MSC isolation and expansion

Mononuclear cells (MNC) were obtained by density gradient centrifugation (Lymphoprep™, Sentinel Ch. Spa, Milan, Italy) of whole CB diluted 1:1 with phosphate-buffered saline (D-PBS, Sigma-Aldrich, St. Louis, MO, USA). MNC were collected from the interphase, washed twice with D-PBS and plated at a density of 1–2 × 10^6^ cells/cm^2^ and 5–7 × 10^6^ cells/ml in low-glucose Dulbecco’s modified Eagle’s medium (DMEM) supplemented with 20% of fetal bovine serum (FBS) (both from Gibco, Thermo Fisher Scientific, Waltham, MA, USA), 10^-7^M dexamethasone (DEXA) (Hospira, Lake Forest, IL, USA), 100 U/ml penicillin and 100 μg/ml streptomycin (Sigma-Aldrich). Cells were then incubated at 37 °C in a humidified atmosphere containing 5% CO_2_ and standard O_2_ concentrations. One week from initial plating, nonadherent cells were removed. Remaining cells were fed once a week and screened for colony appearance for a maximum of 4 weeks (see Additional file 1: Fig. S1). DEXA was added in the culture until the detection of MSC colonies or alternatively supplemented for only the first week of MNC culture (*n* = 16 and *n* = 34 CB units, respectively; see Additional file 2: Fig. S2). MSC colonies at 80% confluence were harvested using 10 × TrypLE Select (Thermo Fisher Scientific) and subcultured at a density of 4000 cells/cm^2^. Standard medium was replaced twice a week and proliferation patterns were established by counting cells each week.

### Growth kinetics and secondary colony-forming ability of CB-MSC

To estimate MSC growth, cells under maintenance conditions were progressively subcultured for 10–12 passages. At each subcultivation, the population doubling (PD) was calculated as follows: PD = log_10_ (N)/log_10_ (2), where N is the number of harvested cells/the number of initially seeded cells. The cumulative PD (cPD) was calculated adding to the PD of the passage under analysis the PDs of the previous passages.

To evaluate the secondary colony-forming ability of CB-MSC, 200 MSC collected at P1 were plated in duplicate into 100-mm diameter culture dishes (Cellstar®, Grainer Bio-One GmbH, Frickenhausen, Germany) for six to seven additional passages. Standard medium was changed weekly and after 2 weeks the cells were fixed with 10% formalin, washed with deionized water and stained with May-Grunwald-Giemsa for 20 minutes. Colonies consisting of at least 30 cells were counted under an inverted light microscope (Axiovert 40 CFL, Zeiss, Oberkochen, Germany).

### Molecular karyotyping

Molecular karyotyping of CB-MSC (*n* = 3) at early (P5) and late passages (P11–13) was performed through array-comparative genomic hybridization (array-CGH) with CytoChip Oligo ISCA 4 × 180 K platform (BlueGnome, Cambridge, UK) and Fluorescent Labelling System (dUTP) kit (BlueGnome). High molecular weight DNA was extracted using the QIAamp DNA Mini kit (Qiagen, Hilden, Germany) according to the manufacturer’s protocol. A pool of characterized genomic DNA (Human Genomic DNA Male and Female, Promega, Madison, WI, USA) was used as control DNA for all experiments. Sample and control DNA were labeled with Cy3 and Cy5 fluorophores, using random primers. Labeling mixes were combined and concentrated for hybridization. Labeled DNA was resuspended with blocking agents in hybridization buffer and applied to the CytoChip Oligo array surfaces using the gasket slides. Hybridization was performed in a rotating oven. Hybridized CytoChips were washed to remove unbound labeled DNA. A laser scanner was used to excite the hybridized fluorophores and read and store the resulting images of the hybridization. Data analysis was performed through BlueFuse Multi for Microarrays v4.0 software-cytochip V2 algorithm (Illumina, San Diego, CA, USA). Quality control parameters for every experiment were evaluated.

### CB-MSC trilineage differentiation

For osteogenic and adipogenic differentiation, CB-MSC at the end of passage 4 were seeded at a density of 4000 cells/cm^2^ on cell culture coverslips (Thermo Fisher Scientific) arranged in 24-well plates (Falcon®, Corning, Corning, NY, USA) in the presence of standard growth medium. At 70–80% of cell confluence, the medium was replaced with specific differentiation media, then renewed every 3–4 days for 21 days. To induce adipogenic differentiation, cells were incubated using the StemPro® Adipogenic Differentiation Kit (Thermo Fisher Scientific), according to the manufacturer’s instructions. The presence of intracellular lipid droplets was detected by standard staining with Oil Red O (Diapath, Bergamo, Italy), according to the manufacturer’s instructions. In parallel, cells were also grown using the StemPro® Osteogenic Differentiation Kit (Thermo Fisher Scientific) to induce osteogenic differentiation. The presence of calcium deposits was evaluated by von Kossa staining (Sigma-Aldrich). Cells were fixed with 10% formalin for 5 minutes at room temperature, incubated with 1% silver nitrate solution for 15 minutes and exposed to ultraviolet light for 2 hours. Coverslips were rinsed with distilled water and 5% sodium thiosulfate to remove unreacted silver. Finally, cells were counterstained with Nuclear Fast Red Solution (Sigma-Aldrich). To induce chondrogenesis, 25 × 10^4^ cells were placed in a 15-ml polypropylene tube (Falcon®, Corning) and washed in order to form a pelleted cellular micromass at the bottom of the tube. The cell pellet was cultured in 500 μl chondrogenic induction medium (StemPro® Chondrogenic Differentiation Kit, Thermo Fisher Scientific), following the recommendations of the manufacturer. Fresh chondrogenic medium was added every 3–4 days. After 28 days, the micromass was fixed, embedded in agar, cut with a microtome and stained with Alcian Blue (Sigma-Aldrich). Cells were counterstained with Nuclear Fast Red Solution.

### RNA isolation and quantitative real-time polymerase chain reaction (qRT-PCR)

Total RNA was extracted using RNeasy Plus Mini Kit (Qiagen) following the manufacturer’s instructions and its quality and quantity were determined using a Nanodrop UV-VIS spectrophotometer (Thermo Fisher Scientific). First-strand cDNA were synthesized from 800 ng of total RNA in 20 μl final volume, using the iScript cDNA synthesis kit (Bio-Rad Laboratories, Hercules, CA, USA) according to the manufacturer’s instructions. The mRNA expression of osteogenic markers RUNX2 and ALP, adipogeneic markers PPARG and FABP4, and chondrogenic markers SOX9 and COLXA1 was quantified by using Sso Fast evaGreen Supermix (Bio-Rad Laboratories) on the ABI 7500 Real-Time PCR System (Applied Biosystems, Thermo Fisher Scientific), according to the producer’s recommendations. Primer sequences are summarized in Additional file 3: Table S1. The thermal cycling protocol involved initial denaturation at 95 °C for 30 sec and was followed by 40 cycles of denaturation at 95 °C for 5 sec and primer annealing and elongation for 32 sec at 60 °C, with a final melting curve analysis to test for the specificity of the product. Data acquisition and analysis were obtained by using SDS v1.4 software (Applied Biosystems, Thermo Fisher Scientific). Each gene was tested in three replicates and three independent experiments were performed. The level of each target gene was normalized to the undifferentiated control by using the 2^-ΔΔCT^ method to quantify the relative changes in gene expression and by applying the efficiency correction represented by the equation: efficiency = 10^(-1/slope)^ -1. TBP and YWHAZ were used as endogenous reference genes [36], provided the verification of their stability under differentiation conditions (Additional file 4: Fig. S3). PCR efficiency corrections were determined for target and reference genes by running a standard PCR curve using diluted cDNA.

### Immunophenotypic analysis

Five color combinations of monoclonal antibodies (mAbs) were used to identify and characterize CB-MSC (*n* = 5) after passage 2 according to the expression of a panel of markers shown in Additional file 5: Table S2. A restricted panel was used to detect the phenotypic modifications induced on MSC by inflammatory priming (see Additional file 6: Table S3). Inflammatory priming was performed by treating CB-MSC at 80% confluence with 10 ng/ml rh-IFN-γ-1b (Imukin, Boehringer-Ingelheim, Ingelheim, Germany) and 15 ng/ml rh-TNF-α (R&D Systems, Minneapolis, MN, USA) for 48 hours of culture, as suggested by the ISCT [37].

About 10^5^ cells were stained for 15 minutes at room temperature in the dark with the specific combination of mAbs. Appropriate fluorescence-minus-one (FMO) and unstained controls were used to determine the level of unspecific binding. At least 10,000 events were acquired on a Cytomics FC500 cytometer (Beckman Coulter, Brea, CA, USA). Data were analyzed by Kaluza software 2.1 version (Beckman Coulter). Expression of individual markers was recorded as the ratio of median fluorescence intensity obtained for each marker and its negative or FMO control in the corresponding fluorescence detector (rMFI).

### Immunomodulation assay

Peripheral blood mononuclear cells (PBMC) were obtained from buffy coats of healthy donors after informed consent. PBMC were isolated by density gradient centrifugation and cryopreserved until use. Thawed PBMC were suspended in RPMI 1640 (Sigma-Aldrich) supplemented with 10% FBS, 1 × L-glutamine (Sigma-Aldrich), 100 U/ml penicillin and 100 μg/ml streptomycin and rested overnight at 37 °C in a humidified atmosphere containing 5% CO_2_ and standard O_2_ concentrations. Overnight resting allowed only a minimal monocyte adhesion, as shown in Additional file 7: Fig. S4. Resting and primed CB-MSC (*n* = 4), the latter stimulated for 48 hours of culture with IFN-γ-1b and TNF-α, were seeded in 96-well flat-bottomed plates (Falcon®, Corning): 4 × 10^4^ cells for the highest (1:0.2) PBMC:MSC ratio were titrated to 1 × 10^4^ to achieve the lowest (1:0.05) PBMC:MSC ratio.

To measure proliferation, PBMC were stained with 5 μM 5,6-carboxyfluorescein diacetate succinimidyl ester (CellTrace™ CFSE Cell Proliferation Kit, Invitrogen, Thermo Fisher Scientific) according to the manufacturer’s instructions. CFSE-labeled cells were seeded on a MSC monolayer at different PBMC:MSC ratios: 1:0.2, 1:0.1, 1:0.05 and 1:0 (no MSC treatment). Cells were stimulated with 0.5 μg/ml of anti-CD3 antibody (Miltenyi Biotec, Bergisch Gladbach, Germany) and 500 UI/ml of recombinant human interleukin-2 (rh-IL-2) (Proleukin®, Novartis, Basel, Switzerland) for 6 days before measuring the corresponding decrease in CFSE fluorescence by flow cytometry. For the latter, anti-human CD45-phycoerythrin-Texas Red (ECD) (J.33 clone, Beckman Coulter) mAb was used to assess proliferation on gated CD45+ cells. At least 50,000 events were acquired on a Cytomics FC500 cytometer. CFSE analysis was performed by Kaluza software and proliferation was quantified as the percentage of cells undergoing at least one cell division.

### Statistical analysis

Clinical information and CB parameters from each donor are presented as relative frequencies or median values and their ranges for each categorical or continuous variable under study. The Kolmogorov-Smirnov and the Shapiro-Wilk tests were used to verify the normal distribution of each continuous variable. The differences between the continuous variables were computed by unpaired *t* test or Mann-Whitney *U* test as appropriate. The differences between categorical variables were computed by Fisher’s exact test. Statistical comparison between resting and primed MSC (i.e., MSC treated or not with inflammatory cytokines) for each MSC batch was performed using the *t* test for matched pairs. Proliferation data are presented as mean with SEM and statistical significance was calculated by two-way ANOVA. *P* values <0.05 were considered statistically significant. Statistical analyses were performed using GraphPad Prism 5.01 software (GraphPad Software Inc., La Jolla, CA, USA).

## Results

### CB-MSC generation

A total of 50 CB units with a median volume of 41 ml (range 18–87 ml) and time after collection of 5.30 h (range 2–24 h) entered this study. MSC isolation was effective in 44% of processed units (22/50). Given the low frequency of MSC progenitors within CB, CB-MSC were mostly isolated as single clones, regardless of the starting volume. MSC colonies were observed at a median of 10.5 days (range 7–20) after MNC plating, while the first trypsinization occurred after a median of 13 days (range 9–22), at about 80% confluence. Differences in either the clinical features of the donors or CB parameters were not globally found between successful and unsuccessful samples, as shown in Table 1.

**Table 1:** Comparison between donor characteristics and successful CB-MSC isolation

	All samples (*n* = 50)	MSC-positive isolation (*n* = 22)	MSC-negative isolation (*n* = 28)	*p* value
Median time after delivery, hours (range)	5 (2–24)	5 (2–24)	7 (2–24)	0.799°
Median TNC × 106 (range)	696 (276–1700)	675 (383–1290)	700 (276–1700)	0.662°
Median MNC × 106 (range)	191 (14–615)	200 (85–615)	191 (14-–62)	0.703°
CB median volume, ml (range)	41 (18–87)	43 (22–87)	40 (18–78)	0.323^§^
Median gestational time, days (range)	273 (259–292)	273 (264–292)	275 (259-–92)	0.288°
Median mother age, years (range)	35 (26–45)	34 (29–41)	35 (26–45)	0.799^§^
Pluriparity	27 (54%)	11 (50%)	16 (57.1%)	0.198*
Male babies	27 (54%)	14 (63.6%)	13 (46.4%)	0.264*
Median baby weight, grams (range)	3390 (2430–4460)	3335 (2700–4460)	3405 (2430-–090)	0.674^§^
Mater/baby blood group match	26 (52%)	15 (68.2%)	11 (39.3%)	0.052*
Cesarean birth	14/48 (29.2%)	7/20 (35%)	7/28 (25%)	0.528*

*Abbreviations: MSC* mesenchymal stromal cells, *TNC* total nucleated cells, *MNC* mononuclear cells, *CB* cord blood

Statistical tests:

°Mann-Whitney *U* test

^§^Unpaired *t* test

*The differences between the categorical variables were computed by the Fisher exact test

### Effect of dexamethasone exposure on CB-MSC culture outgrowths

As first approach we cultured 16 CB units in the presence of 10^-7^ M DEXA until the detection of MSC growing colonies [34]. CB-MSC clones were isolated from 37.5% CB units (6/16). Colonies were detected at a median of 12.5 days from initial plating (range 8–20) and harvested after a median of 13.5 days (range 13–22). All samples except one reached at least five passages.

To assess whether a lower exposure to DEXA could improve CB-MSC isolation and proliferation capability, a second series of CB units (*n* = 34) was subjected to DEXA supplementation for the first week of MNC culture only. In this condition, MSC isolation was successful in 47.1% units (16/34), with a median detection and harvest time of 10 (range 7–15) and 12 days (range 9–15), respectively. All samples were capable to reach at least five passages.

**Fig. 1:**
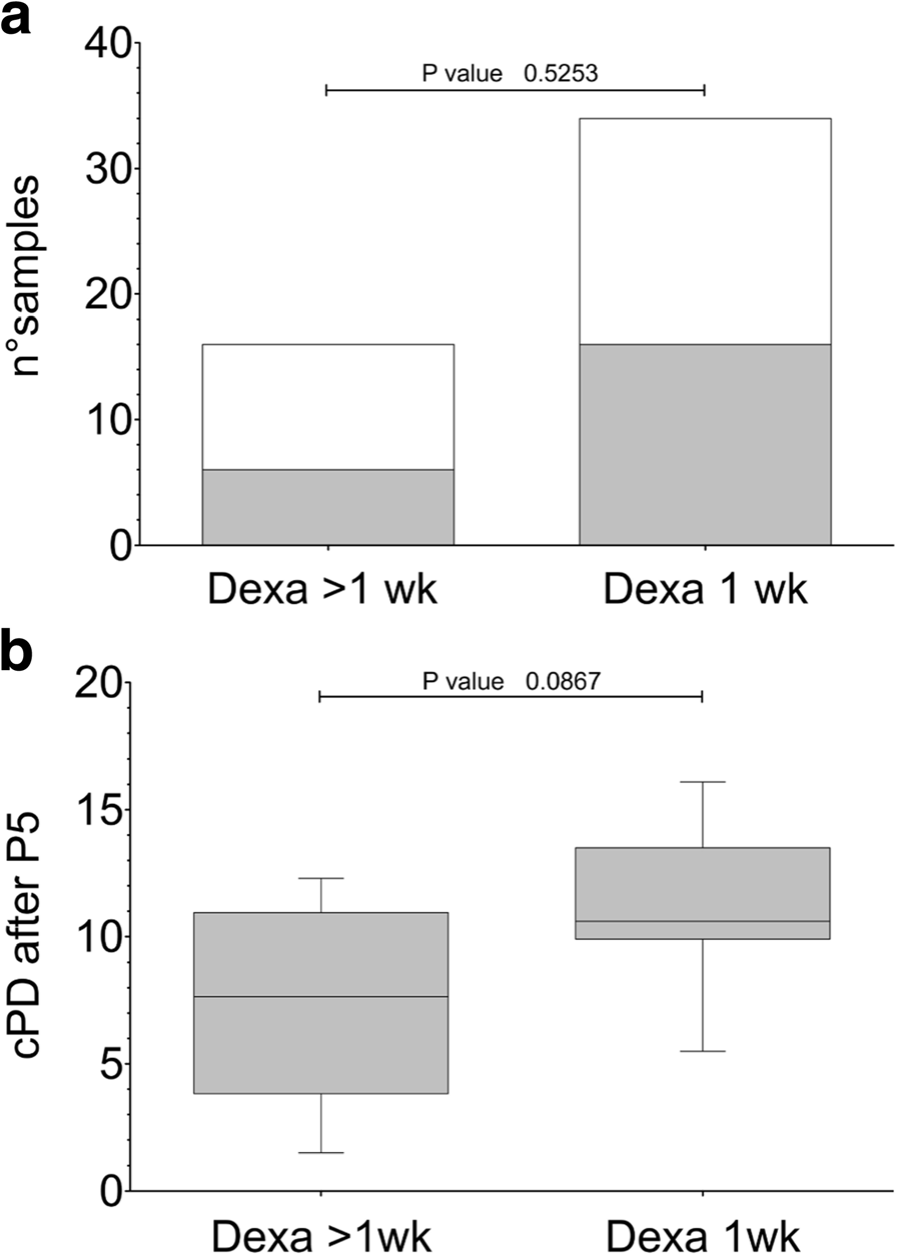
Effect of dexamethasone on CB-MSC culture outgrowths. **a** Effects of two different treatment regimens with DEXA (>1 wk or 1 wk, *n* = 16 and *n* = 34, respectively) on CB-MSC isolation (*n* = 6 and *n* = 16, respectively). *Gray color*: positive MSC isolation. *White color*: negative MSC isolation. The differences were computed by Fisher exact test, *p* > 0.05. **b** Comparison of cumulative population doubling (cPD) at P5 between CB-MSC isolated by adding DEXA for > 1wk or 1 wk (*n* = 6 and *n* = 15, respectively). The differences were computed by Mann-Whitney *U* test, *p* > 0.05. Boxes extend from 25^th^ percentile to the 75^th^ percentile, the middle line represents median value and the whiskers extend from minimum to maximum values. *Abbreviations: cPD* cumulative population doublings, *DEXA* dexamethasone, *wk* week

The withdrawal of DEXA after the first week of MNC culture did not significantly modify either the efficiency of CB-MSC isolation (*p* = 0.5253, Fig. 1a) or the cPD at P5 (*p* = 0.0867, Fig. 1b).

### CB-MSC growth characteristics

**Fig. 2:**
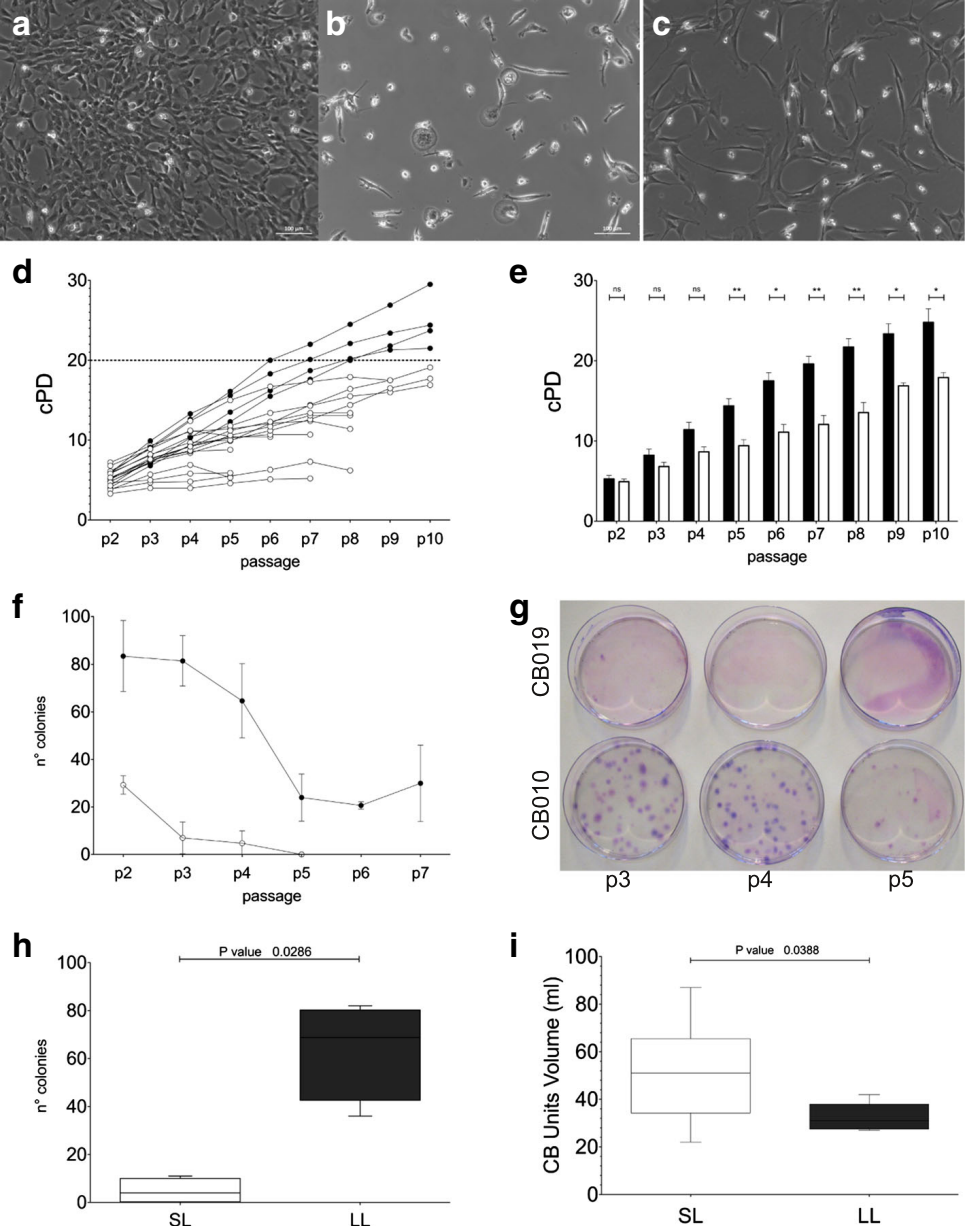
Morphology and growth characteristics of CB-MSC. **a** Colony of CB-MSC 10 days after initial seeding (passage 0). **b** Non-proliferative fibroblast-like cells and osteoclast-like cells, the latter with very large cytoplasm and occasional multiple nuclei (passage 0). **c** Morphology of CB-MSC at passage P1. Scale bars: 100 μM. **d** Growth patterns of CB-MSC grouped by similar cPD (cPD cutoff = 20 at P9). *Black circles*: LL-CBMSC; *white circles*: SL-CBMSC. **e** Comparison of cPD between LL- (*black bars*) and SL- (*white bars*) CBMSC at each passage; the differences were computed by Mann-Whitney *U* test, ^*^ *p* < 0.05, ^**^ *p* < 0.01, ^***^ *p* < 0.001; data are presented as mean with SEM. **f** Secondary colony formation of LL-CBMSC (*black circles*) and SL-CBMSC (*white circles*) at defined passages. **g** Colonies formed after plating 200 MSC in 100-mm culture dishes are shown from one representative LL- and one SL-CBSMC (CB010 and CB019, respectively). **h** Secondary colony formation of LL-CBMSC (*black boxes*) and SL-CBMSC (*white boxes*) at P4. The differences were computed by Mann-Whitney *U* test, *p* < 0.05. Boxes extend from 25^th^ percentile to the 75^th^ percentile, the middle line represents median value and the whiskers extend from minimum to maximum values. **i** Comparison between CB volumes between LL-CBMSC and SL-CBMSC (*n* = 5 and *n* = 16, respectively); the differences were computed by Mann-Whitney *U* test, *p* < 0.05. *Abbreviations: LL-CBMSC* long-living CBMSC, *SL-CBMSC* short-living CBMSC, *NS* not significant

The isolated CB-MSC displayed initially a small spindle-shape morphology and a high degree of heterogeneity, mainly due to the contamination by osteoclast-like cells and non-proliferating fibroblast-like cells. These contaminating cells that were strongly adhered to the bottom of the flasks were eliminated by P2 passage (Fig. 2a-c).

Differences in the proliferative capacity and exhaustion passage were observed between MSC from different units. Overall, 1/3 of CB-derived MSC were able to expand for more than nine passages. By evaluating the long-term proliferative potential at least two growth kinetics patterns were recognized. We distinguished short- and long-living (SL- and LL-) CB-MSC based on their lower or higher cPD, respectively (cPD cutoff = 20 at p9). LL-CBMSC displayed a constant greater growth and longevity than SL-CB-MSC (Fig. 2d). Moreover, by comparing the cPD at each passage, significant differences in the proliferative capacity were revealed by passage 5 (Fig. 2e).

Since the discrimination between SL- and LL-CBMSC based on the cPD could only be done retrospectively, we sought to identify an earlier distinctive marker, possibly of clinical utility for the choice of the batches of CB-MSC suitable for large-scale expansion and clinical use. As already demonstrated, the heterogeneous proliferative potential reflected differences in the self-renewal capacity [21, 38]. By assessing the secondary colony-forming capability of the two populations, we found that LL-CBMSC retained greater secondary colony-forming ability compared to SL-CBMSC. Conversely, SL-CBMSC failed to self-renew after a few passages then lost the growth capacity earlier (Fig. 2f-g). Significant differences were specifically observed at passage 4, albeit on a limited number of samples (Fig. 2h).

We next addressed the role of donor characteristics and CB parameters (listed in Table 1) as discriminating markers between LL- and SL-CBMSC. Quite surprisingly, we found that the median of CB volumes of units giving rise to SL-CBMSC was significantly higher (51 ml, range 22–87) with respect to the volume of CB units giving rise to LL-CBMSC (31 ml, range 27–42) (*p* = 0.0388, *n* = 16 and *n* = 5, respectively, Fig. 2i).

Finally, in order to test the genetic stability of CB-MSC after prolonged expansion, three LL-CBMSC batches at early (P5) and late passages (P11–13) were tested for their genomic assets through array-CGH analysis. Results revealed that expanded CB-MSC did not show unbalanced chromosomal rearrangements (deletion or duplication), excluding copy number variation constitutionally present (see Additional file 8: Fig. S5).

### Multilineage differentiation

**Fig. 3:**
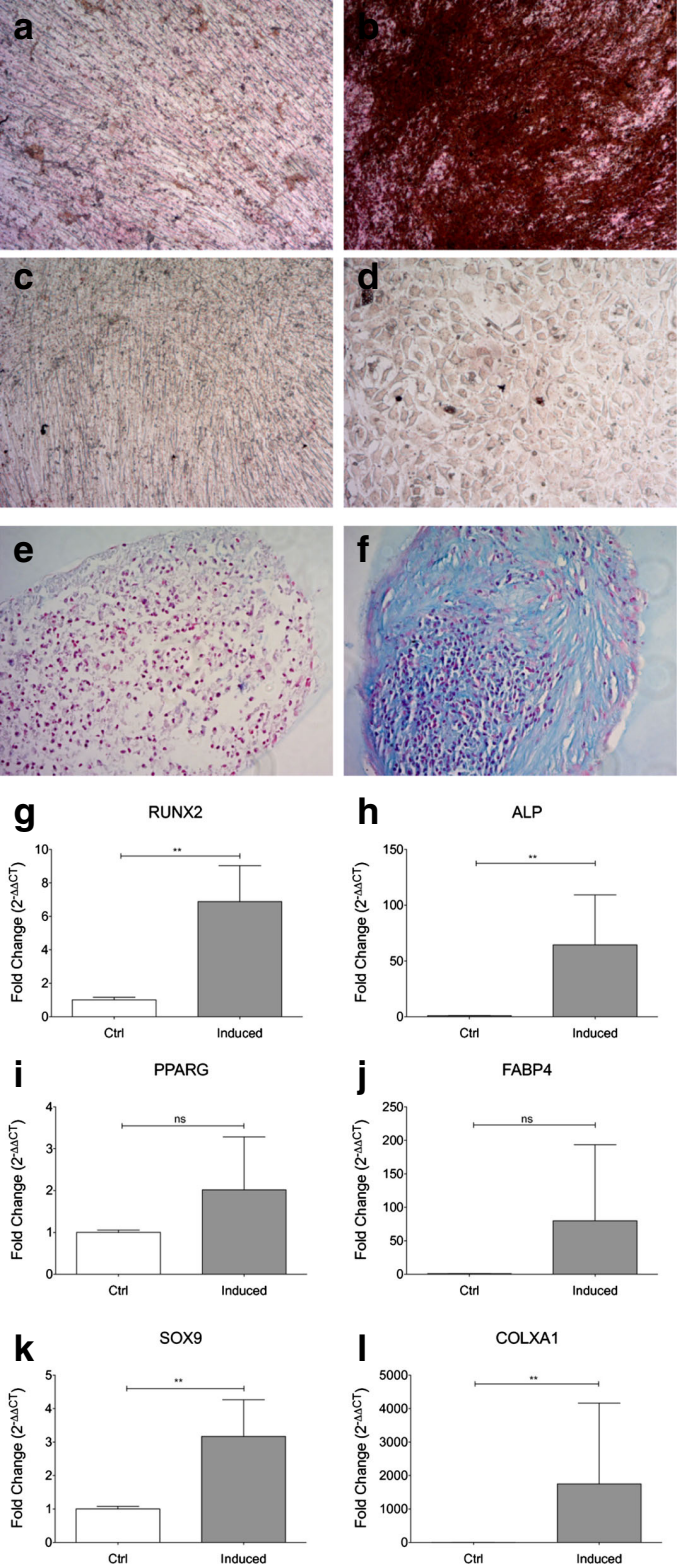
Multilineage differentiation of CB-MSC. Multilineage ability was determined in P4 LL-CBMSC. **a**-**f** Panels display cells which have been induced to differentiate in vitro toward osteogenic (**a**-**b**), adipogenic (**c**-**d**), and chondrogenic (**e**-**f**) lineages. Osteogenic and adipogenic differentiation were assessed after 21 days of induction using von Kossa and Oil Red O staining, respectively; ×10 magnification. Chondrogenesis was evaluated by Alcian Blue staining at day 28 of induction; cells were counterstained with Nuclear Fast Red solution; ×20 magnification. For each staining, undifferentiated controls are also displayed on the *left* (panels **a**-**c**-**e**). **g** Quantitative RT-PCR analysis of osteogenic markers RUNX2 and ALP (**g**-**h**), adipogenic markers PPARG and FABP4 (**i**-**j**), and chondrogenic markers SOX9 and COLXA1 (**k**-**l**) in cells cultured under the respective lineage induction conditions. Results are presented as the fold change in mRNA expression in respect to TBP as representative reference gene and to the undifferentiated control. The mean values from three independent experiments done in triplicate are shown. The differences were computed by paired *t* test or Wilcoxon matched pairs test as appropriate, *p* values: ^**^ *p* < 0.01. *Abbreviations: NS* not significant

To investigate the in vitro differentiation potential of CB-MSC from various LL donors, cells at P4 were induced to differentiate down the osteogenic, adipogenic and chondrogenic lineages, by using defined media components and culture conditions (Fig. 3a-f). All CB-MSC (*n* = 5) demonstrated osteogenic differentiation after 3 weeks of induction. By contrast, we observed poor adipogenic potential (1/5 samples) as revealed by Oil Red O staining. When cultured under chondrogenic conditions, cartilage-like cells with lacunae and a large amount of cartilage extracellular matrix were observed in sections of pellets from all samples. Parallel experiments on SL-CBMSC confirmed the absence of dissimilarities compared to LL-CBMSC in regard to osteogenic and adipogenic multilineage differentiation (Additional file 9: Fig. S6), while chondrogenic potential was not assessed due to the difficulty to obtain a sufficient number of SL cells for the assay.

To confirm multilineage differentiation at a molecular level, the transcript levels of both early- and late-stage markers of adipogenesis, osteogenesis, and chondrogenesis were determined by means of qRT-PCR in LL-CBMSC. Results from three independent experiments confirmed, even with variability between MSC donors, significant upregulation of all mRNA transcripts involved in chondrogenic and osteogenic MSC differentiation (*p* = 0.0039 for SOX9, RUNX2, and ALP; *p* = 0.0078 for COLXA1), while the absence of significant differences for the adipogenic markers PPARG and FABP4 (*p* = 0.0547) (Fig. 3i-j).

### Immunophenotypic analysis

**Fig. 4:**
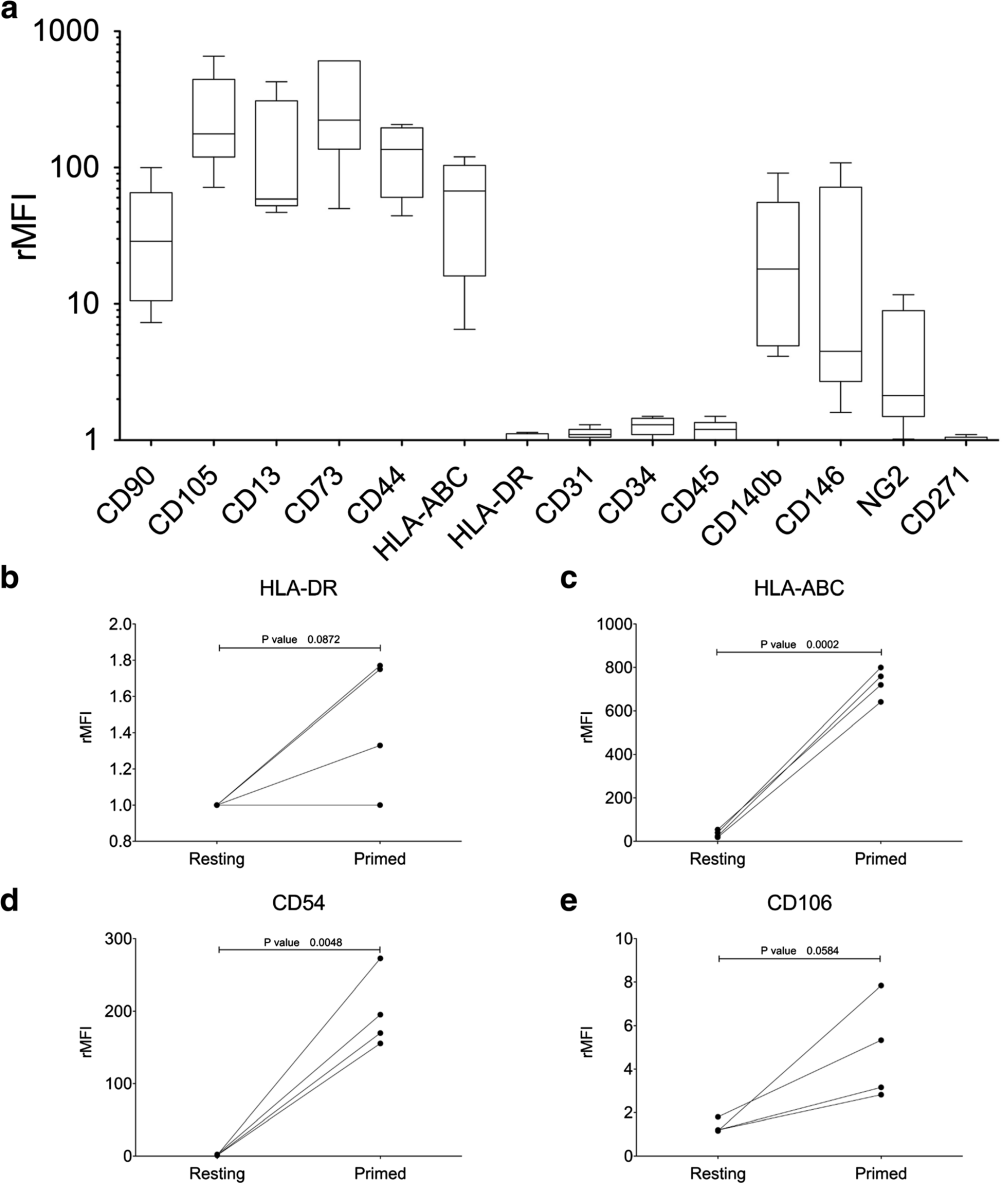
Immunophenotypic analysis of CB-MSC. **a** Characterization of LL-CBMSC (*n* = 5) by flow cytometry using a panel of 14 cell surface markers. Boxes extend from 25^th^ percentile to the 75^th^ percentile, the middle line represents median value and the whiskers extend from minimum to maximum values. Data are displayed as rMFI on the unstained control. **b**-**e** Phenotypic modifications induced on LL-CBMSC (*n* = 4) by inflammatory stimuli, i.e., treatment with 10 ng/ml IFN-γ-1b and 15 ng/ml TNF-α for 48 hours before staining with the appropriate mAb combination. Data are expressed as rMFI with respect to the FMO control. *P* values <0.05 were considered statistically significant. *Abbreviations: rMFI* relative median fluorescence intensity, *FMO* fluorescence-minus-one, *mAb* monoclonal antibody

Immunophenotypic characterization was performed by flow cytometry in agreement with ISCT criteria. Relevant MSC-related and pericyte markers were investigated based on current literature [39]. Culture-expanded LL-CBMSC (*n* = 5) strongly expressed the MSC markers CD90, CD105, CD44, CD13, and HLA-ABC, while they were negative for the hematopoietic markers CD31, CD34, CD45, and for HLA-DR. Additional markers searched for on the MSC surface showed variable expression, such as the perivascular antigens PDGFRβ, CD146, and NG2 (Fig. 4a). As already reported by other authors, CB-MSC were found negative for CD271 [20, 40]. None of the investigated markers was found differentially expressed on the surface of LL- compared to SL-CBMSC (Additional file 10: Fig. S7).

We then evaluated the modifications of MSC immunophenotype after treatment with IFN-γ-1b and TNF-α for 48 hours, corresponding to induction of immunosuppressive function in MSC [5]. As previously demonstrated, the expression of HLA-ABC, HLA-DR, CD54 (ICAM-1), and CD106 (VCAM-1) was modulated in the presence of inflammatory priming [40]. Particularly, significant upregulation was observed for CD54 (low-negative at resting conditions) and HLA-ABC (high-positive at resting conditions) (*p* = 0.004 and *p* < 0.001, respectively, Fig. 4 c-d). Upregulation of CD106 (low-negative at resting conditions) did not reach significance, while the expression of HLA-DR (negative in resting MSC) was almost unchanged (Fig. 4b-e).

### Immunosuppressive properties of CB-MSC

**Fig. 5:**
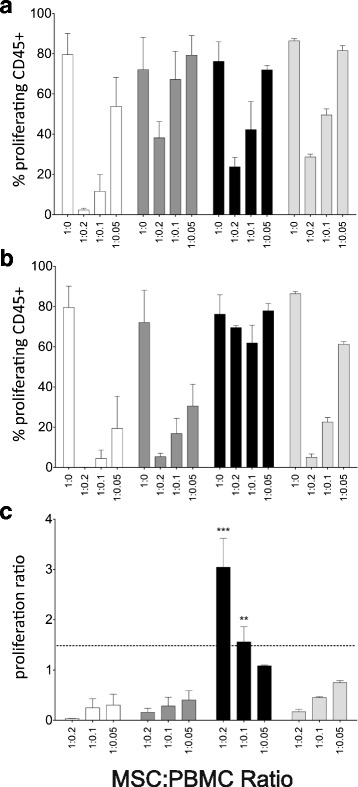
Immunosuppressive properties of CB-MSC. a-b Inhibitory effect of resting (**a**) and primed (**b**) LL-CBMSC on allogeneic CFSE-labeled PBMC. Cells were co-cultured at different PBMC:MSC ratios upon PBMC stimulation with anti-CD3 and rh-IL-2 for 6 days. Three different PBMC:MSC ratios were used ranging from 1:0.2 to 1:0.05. The 1:0 ratio represents the positive control (no MSC treatment and presence of PBMC antibody stimulation). Proliferation was assessed by CFSE dilution method on CD45+ cells. Each bar represents mean and SEM of two independent experiments with two different PBMC donors for each of four CB-MSC batches. **c** Proliferation ratio between the percentage of CD45+ proliferation at primed and resting conditions, at the different MSC doses. The differences were computed by two-way ANOVA, ^**^ *p* < 0.01, ^***^ *p* < 0.001

MSC are known for their remarkable ability to suppress the proliferation of several immune cell types [2]. We tested the immunosuppressive properties of CB-MSC (specifically LL-CBMSC) by assessing their capacity to modulate the proliferative response of CFSE-labeled PBMC upon stimulation with anti-CD3 and rh-IL-2. MSC batches (*n* = 4) at P5-P6 were analyzed, provided with additional experiments that there were no differences in the inhibitory potential with passaging (e.g., from P2 to P6) on both resting and primed MSC (Additional file 7: Fig. S4). Flow cytometry analysis of CFSE dilution on CD45+ cells showed that proliferation of activated PBMC was generally suppressed by MSC in a dose-dependent manner (Fig. 5a-b). Nevertheless, significant differences in the inhibitory potential were revealed between individual MSC batches, particularly after IFN-γ-1b and TNF-α priming (Fig. 5b). We thus expressed the MSC inhibitory potential in terms of proliferation ratio, as the ratio between the percentage of CD45+ proliferation at primed and resting conditions. In most cases, the proliferation ratio increased inversely with MSC dose. For only one CB-MSC batch, a proliferation ratio directly increasing with MSC dose was observed, suggesting the lack of inhibition by inflammatory-primed MSC on PBMC proliferation (Fig. 5c). In this case, the proliferation ratio was found significantly greater with respect to other batches, specifically at 1:0.2 and 1:0.1 PBMC:MSC ratio (*p* < 0.001 and *p* < 0.01, respectively, Fig. 5c).

## Discussion

Obtaining definitive data on the effectiveness of MSC in the clinic is hampered by the lack of standardized protocols used to prepare large-scale MSC and of useful tests to compare their potency. Particularly, differences in donor source, culture methods, and expansion levels are critical in determining MSC functionality [41, 42].

Our study investigated whether CB may represent a suitable source of MSC for cell-based therapeutic strategies. Furthermore, the biological and functional properties of CB-derived MSC were assessed in view of a more effective and safer clinical use. By applying quality criteria for an optimal CB-MSC isolation (CB volume ≥ 20 ml, time from collection ≤ 24 h), we were able to isolate MSC colonies from 44% of processed units. We next evaluated whether isolation was influenced by any clinical features of the donors or CB parameters, but we found no correlation between the analyzed parameters and the rate of success in isolating CB-MSC. Other studies reported isolation yields ranging from fewer than 10% to 90%, revealing a lack of consensus in the methodological approaches and selection criteria for CB units [20, 21, 29, 30, 43]. By using DEXA (10^-7^ M) as medium supplement in addition to 20% FBS for 1 week, Zhang et al. achieved a 90% rate of success in isolating CB-MSC when the volume was ≥ 90 ml and the time to processing ≤ 2 h [20]. By applying the same criteria, Pievani et al. were able to obtain MSC from 40% of processed units only [35].

DEXA was found to inhibit monocyte adhesion, thus it is conceivable a role of the steroid in supporting the proliferation of CB-MSC progenitors at the expense of other contaminants which can adhere on culture plates. Therefore, its supplementation was applied also in our study with the aim to promote the adhesion of the rare CB-MSC progenitors to the plastic. Moreover, Zhang and co-workers found no benefit from using alternative isolation methods or culture conditions, such as immunodepletion before MNC plating, addition of growth factors to the standard MSC medium, culture in hypoxia, or coating strategies [20]. Even though the use of DEXA has been described in the generation of CB-MSC [20, 34, 35], its dosage varied between investigators. In the first isolation protocol published by Kögler et al. [34], DEXA (10^-7^ M) was added until the detection of MSC growing colonies, while the expansion was performed in the absence or at a low concentration of the supplement. In the present study, the choice to evaluate two treatment regimens with DEXA was of particular significance in order to define a standardized protocol for generating CB-MSC for clinical use. Our results showed that the extent of DEXA supplementation did not significantly affect CB culture outcomes, in regard to both isolation efficiency and long-term proliferation, even if there seemed to be better outcomes in the group of units treated with DEXA for only 1 week. Given that culture conditions may have remarkable effects on the functionality of MSC [33, 44], the addition of uncommon supplements to standard MSC medium, like hormones, should be avoided or minimized, at least when the purpose is to produce MSC with unaltered stemness properties for clinical use. It should be therefore more appropriate and equally effective keeping cell exposure to DEXA to the minimum required for an effective CB-MSC isolation.

As discussed, there are some drawbacks linked to the successful isolation and expansion of CB-MSC, mainly due to the low frequency of MSC clones in particular when compared with umbilical cord (UC), a rich source of high-proliferative MSC characterized by isolation yields of 100% [45]. To the other hand, UC is a heterogeneous tissue whose processing is time-consuming and labor-intensive in respect to the easier manipulation of CB. Moreover, public CB banks provide an easy-to-access system for using freshly donated CB units for MSC generation, while a similar collection network system does not exist for UC or other fetal sources like placenta. The present study has also revealed that the role of volume as selection criterion for CB units processed for CB-MSC isolation should be re-considered because of affecting neither the rate of successful MSC isolation nor the growth potential. This finding would allow to use even CB units of low volume for MSC isolation.

An important outcome of the present study was to analyze several in vitro parameters that may help to better define the “quality” of CB-derived MSC prior to their clinical application. The evaluation of the proliferation capacity as first indicator of MSC potential allowed us to corroborate the findings from Barilani and co-authors [21], who demonstrated the existence of at least two stromal populations within CB, one long-living (LL-CBMSC) and the other short-living (SL-CBMSC) on the basis of their proliferative ability, secondary colony-forming efficiency and most importantly, telomere length. The same authors found LL-CBMSC mostly indiscernible from the SL counterpart in regard to ISCT criteria [32]. We confirmed that the two MSC populations clearly diverge in their growth capacity and secondary colony-forming efficiency, thus suggesting a potential role of these in vitro parameters as indicators of CB-MSC longevity. On one hand, the possibility of cryopreserving low-passage CB-MSC batches in advance in respect to the clinical needs should allow to monitor cell growth until the established cPD cutoff, thus rendering the retrospective discrimination between LL- and SL-CBMSC clinically useful. On the other hand, the evaluation of secondary colony formation may be a promising and likely early parameter of longevity.

Our data confirmed the absence of major dissimilarities concerning immunophenotype and multilineage differentiation, fitting both populations the minimal panel proposed for MSC definition [1]. It is conceivable that LL-CBMSC represent better candidates for obtaining clinically relevant numbers of MSC for therapeutic applications, due to the higher ex vivo expansion and the ability to survive for longer periods in vitro. Therefore, the assessment of their genetic stability after prolonged culture represents an important release criterion for a safe clinical use, even if overly expansion should be avoided too [46, 47].

Unlike MSC from other sources, is widely reported the relatively low adipogenesis ability of CB-MSC under standard induction protocols [48–50], most likely due to their more primordial, fetal origin in respect to other MSC sources [51]. On the other hand, it is well documented that there is more propensity toward chondrogenesis and osteogenesis in vitro under appropriate culture conditions and to some extent in vivo [20, 35, 52, 53]. Sacchetti and co-workers [53] have recently demonstrated a unique capacity of CB-derived stromal cells to form cartilage in vivo spontaneously, in addition to an osteogenic capacity. Therefore, they support the presence within CB of chondro-osteoprogenitors rather than multipotent MSC, whose origin, however, remains to be elucidated. Our data confirmed an impaired adipogenic potential of CB-MSC, revealed by a clear absence of cells forming lipid droplets and confirmed at a molecular level by the absence of significant mRNA induction of PPARG and FABP4, the former representing a crucial player during the transcriptional cascade leading to adipogenic differentiation, the latter being its direct target. More in general, our molecular analysis highlighted a wide variability in the differentiation potential between individual samples. This finding may fit the increasing evidence of the existence of distinct CB-derived stromal progenitors, possibly of different developmental origin and related plasticity [53–56]. In this regard, unrestricted somatic stromal cells (USSC) and cord blood-derived stromal cells (CB-MSC) were originally defined according to the expression of the adipogenic inhibitor delta-like 1 (DLK-1), a specific marker of USSC correlating with a lack of adipogenic differentiation ability and a higher proliferative potential compared to the more differentiated and less proliferative CB-MSC [54]. Even if in our experience a lack of adipogenic potential was common to both LL- and SL-CBMSC, it remains unclear whether the more immature USSC match our LL-CBMSC.

The ultimate aim of the present work was to investigate the immunosuppressive activity of both resting and inflammatory-primed CB-MSC, in order to complete their functional characterization prior to clinical application. Despite the recent suggestions provided by ISCT [37, 42, 57], a universally accepted in vitro method to assess MSC immunosuppression does not exist [58, 59]. We used unselected and opportunely stimulated PBMC because more closely mimic the in vivo inflammatory environment to which MSC are exposed on patient administration. Our results showed that CB-MSC inhibited PBMC proliferation with different efficacy, particularly after treatment with exogenous IFN-γ-1b and TNF-α. The lack of efficacy of one batch was found despite the flow cytometry upregulation of priming-inducible markers. Moreover, it was confirmed repeatedly on different PBMC donors. In this situation, a trophic effect mediated by primed CB-MSC was conceivable on PBMC proliferation. Additional studies would be warranted to determine how correlating this observation with other immunomodulatory markers, for adequately predicting the in vivo potency of CB-MSC. von Bahr and co-authors [60] reported no evidence of correlation between the in vitro inhibitory potential of MSC and in vivo clinical response in patients with aGvHD, suggesting that the in vivo efficacy of MSC not only depends on the intrinsic properties of the MSC preparation but also on the product-host interaction. A detailed immunomonitoring of patients as well as an in-depth characterization of MSC would therefore be helpful to correlate the in vitro measure of the potency with clinical outcomes [61].

## Conclusions

In summary, the present work shows that CB may be a practical source of MSC for clinical applications. CB remains much more readily available and devoid of major ethical concerns in respect to other conventional MSC sources. To the best of our knowledge, we have provided evidence that not all LL-CBMSC are equally immunosuppressive in an inflammatory environment, in support of the need to include the assessment of in vitro potency among the release criteria for each CB-MSC batch intended for clinical use, at least for the treatment of immune disorders such as GvHD.

## Additional files

Additional file 1: Figure S1. Schematic protocol for CB-MSC generation. Success or failure in isolation of CB-MSC was assessed by the appearance of colonies to a maximum of 4 weeks from MNC plating. *Abbreviations: MNC* mononuclear cells. (DOCX 104 kb)
Additional file 2: Figure S2. Dexamethasone scheduling in MNC culture. The supplement was added in standard medium until the detection of MSC colonies (*n* = 16 CB units) or alternatively added for the first week of MNC culture only (*n* = 34). (DOCX 120 kb)
Additional file 3: Table S1. Genes and primer sequences used for quantitative real-time PCR (RT-PCR). (DOCX 13 kb)
Additional file 4: Figure S3. Stability of endogenous reference genes (TBP and YWHAZ) under differentiation conditions. Quantitative RT-PCR analysis of TBP normalized with respect to YWHAZ (A-C) and vice versa (D-F) under osteogenic (A and D), chondrogenic (B and E) and adipogenic (C and F) differentiation conditions. Results are presented as the fold change in mRNA expression obtained by using the 2^-ΔΔCT^ method, applying the correction efficiency for each gene. The mean values from three independent experiments done in triplicate are shown. The differences were computed by Wilcoxon matched pairs test, *p* > 0.05. *Abbreviations: NS* not significant. (DOCX 526 kb)
Additional file 5: Table S2. List of antibodies used for immunophenotypic analysis. (DOCX 14 kb)
Additional file 6: Table S3. Five-color mAb combinations used for immunophenotypic analysis of inflammatory MSC priming. (DOCX 14 kb)
Additional file 7: Figure S4. Immunomodulation assay. Flow cytometry analysis of thawed PBMC before (A) and after (B) overnight resting. The percentage of monocytes of one representative sample is shown. (C) Proliferation ratio between the percentage of CD45+ proliferation at primed and resting conditions, at the different MSC passages. Results from two independent experiments against two different PBMC lots are shown. (DOCX 637 kb)
Additional file 8: Figure S5. Representative array-CGH profiles of the whole genome of one representative LL-CBMSC sample at P5 (*lower panel*) and P11 (*upper panel*) (DOCX 107 kb)
Additional file 9: Figure S6. SL-CBMSC multilineage differentiation. Panels display cells which have been induced to differentiate in vitro toward osteogenic (A-B) and adipogenic (C-D) lineages. Osteogenic and adipogenic differentiation were assessed after 21 days of induction using von Kossa and Oil Red O staining, respectively; ×10 magnification. For each staining, undifferentiated controls are also displayed on the *left* (panels A-C). One representative sample is shown. (DOCX 2.42 mb)
Additional file 10: Figure S7. SL-CBMSC immunophenotypic analysis. Characterization of SL-MSC (*n* = 6) by flow cytometry using a panel of 14 cell surface markers. Boxes extend from 25^th^ percentile to the 75^th^ percentile, the middle line represents median value and the whiskers extend from minimum to maximum values. Data are displayed as rMFI on the unstained control. (DOCX 104 kb)

## Abbreviations

aGvHD: acute graft-versus-host disease
BM: bone marrow
CB: cord blood
CFSE: carboxyfluorescein diacetate succinimidyl ester
CGH: comparative genomic hybridization
cPD: cumulative population doublings
DEXA: dexamethasone
DMEM: Dulbecco’s modified Eagle’s medium
ECD: phycoerythrin-Texas Red
FBS: fetal bovine serum
FMO: fluorescence-minus-one
HSCT: hematopoietic stem cell transplantation
IFN-γ-1b: interferon-gamma-1b
ISCT: International Society for Cellular Therapy
LL-CBMSC: long-living cord blood mesenchymal stromal cells
mAb: monoclonal antibody
MNC: mononuclear cells
MSC: mesenchymal stromal cells
PBMC: peripheral blood mononuclear cells
PBS: phosphate-buffered saline
qRT-PCR: quantitative real-time polymerase chain reaction
rh-IL-2: recombinant human interleukin-2
rMFI: relative median fluorescence intensity
SL-CBMSC: short-living cord blood mesenchymal stromal cells
TNC: total nucleated cells
TNF-α: tumor necrosis factor alpha
UC: umbilical cord
USSC: unrestricted somatic stromal cells
