# Supplementary material for: Generation of mesenchymal stromal cells from cord blood: evaluation of in vitro quality parameters prior to clinical use
Source: Stem Cell Res Ther. 2017 Jan 24;8:14. doi: 10.1186/s13287-016-0465-2 (PMC5260040; doi:10.1186/s13287-016-0465-2)
Supplement: Additional file 1: Figure S1. — Schematic protocol for CB-MSC generation. Success or failure in isolation of CB-MSC was assessed by the appearance of colonies to a maximum of 4 weeks from MNC plating. Abbreviations: MNC mononuclear cells. (DOCX 104 kb) [file 13287_2016_465_MOESM1_ESM.docx]

**Additional file 1**

**Figure S1: Schematic protocol for CB-MSC generation.** Success or failure in isolation of CB-MSC was assessed by the appearance of colonies to a maximum of 4 weeks from MNC plating. Abbreviations: MNC, mononuclear cells.

**
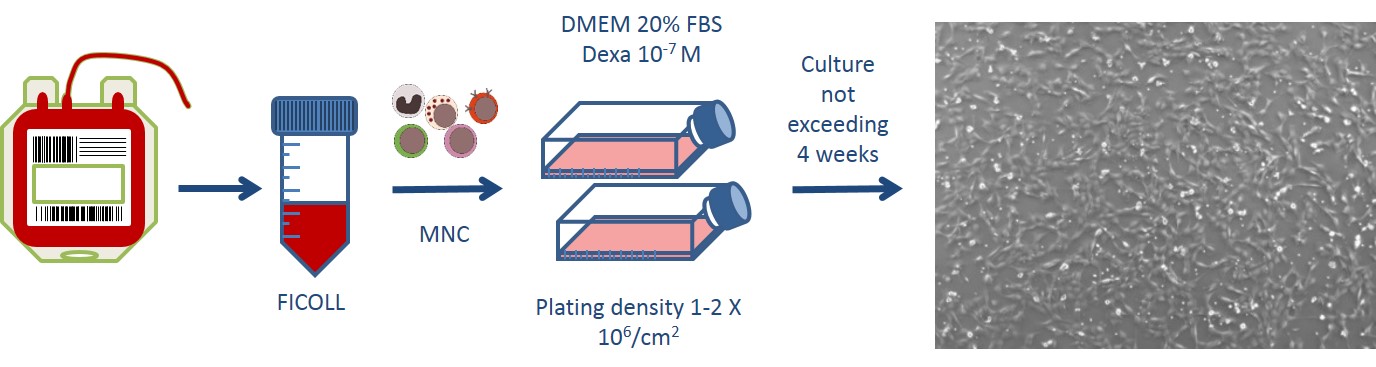
**
