# Supplementary material for: Generation of mesenchymal stromal cells from cord blood: evaluation of in vitro quality parameters prior to clinical use
Source: Stem Cell Res Ther. 2017 Jan 24;8:14. doi: 10.1186/s13287-016-0465-2 (PMC5260040; doi:10.1186/s13287-016-0465-2)

**Additional file 2**

**Figure S2: Dexamethasone scheduling in MNC culture.** The supplement was added in standard medium until the detection of MSC colonies (n=16 CB units) or alternatively added for the first week of MNC culture only (n=34).


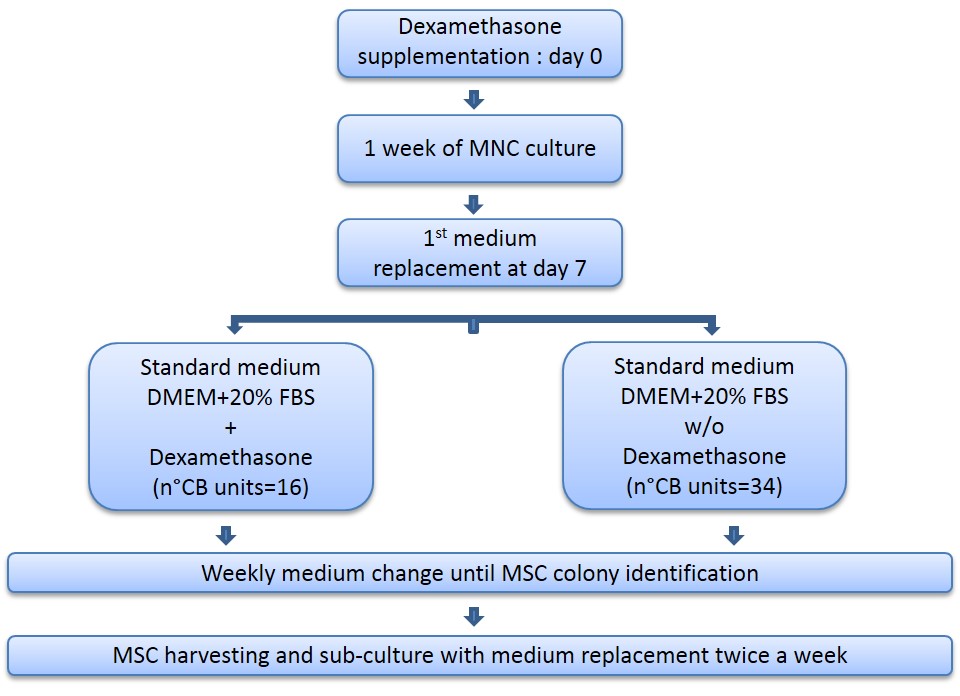

Supplement: Additional file 2: Figure S2. — Dexamethasone scheduling in MNC culture. The supplement was added in standard medium until the detection of MSC colonies (n = 16 CB units) or alternatively added for the first week of MNC culture only (n = 34). (DOCX 120 kb) [file 13287_2016_465_MOESM2_ESM.docx]
