# Supplementary material for: Generation of mesenchymal stromal cells from cord blood: evaluation of in vitro quality parameters prior to clinical use
Source: Stem Cell Res Ther. 2017 Jan 24;8:14. doi: 10.1186/s13287-016-0465-2 (PMC5260040; doi:10.1186/s13287-016-0465-2)
Supplement: Additional file 3: Table S1. — Genes and primer sequences used for quantitative real-time PCR (RT-PCR). (DOCX 13 kb) [file 13287_2016_465_MOESM3_ESM.docx]

**Additional file 3**

**Table S1:** Genes and primer sequences used for quantitative real time PCR (qRT-PCR).

TBP

F: GCCACGCCAGCTTCGGAGAG

R: CCGCAGCAAACCGCTTGGGA

YWHAZ

F: CCGCTGGTGATGACAAGAAAGGGAT

R: AGGGCCAGACCCAGTCTGATAGGA

SOX9

F: CTGGGCAAGCTCTGGAGACTTCTG

R: TTCACCGACTTCCTCCGCCG

PPARG

F: CATTCCATTCACAAGAACAGAT

R: GGCTTATTGTAGAGCTGAGT

ALP

F: TACAAGGTGGTGGGCGGTGAACGA

R: TGGCGCAGGGGCACAGCAGAC

COLXA1

F: ACTCCCAGCACGCAGAATCCA

R: TGGGCCTTTTATGCCTGTGGGC

FABP4

F: ATGGGATGGAAAATCAACCA

R: GTGGAAGTGACGCCTTTCAT

RUNX2

F: AAGGCTGCAAGCAGTATTTACAA

R: CTCGGATCCCAAAAGAAGTTTTGCT
