# Supplementary material for: Generation of mesenchymal stromal cells from cord blood: evaluation of in vitro quality parameters prior to clinical use
Source: Stem Cell Res Ther. 2017 Jan 24;8:14. doi: 10.1186/s13287-016-0465-2 (PMC5260040; doi:10.1186/s13287-016-0465-2)
Supplement: Additional file 4: Figure S3. — Stability of endogenous reference genes (TBP and YWHAZ) under differentiation conditions. Quantitative RT-PCR analysis of TBP normalized with respect to YWHAZ (A-C) and vice versa (D-F) under osteogenic (A and D), chondrogenic (B and E) and adipogenic (C and F) differentiation conditions. Results are presented as the fold change in mRNA expression obtained by using the 2-ΔΔCT method, applying the correction efficiency for each gene. The mean values from three independent experiments done in triplicate are shown. The differences were computed by Wilcoxon matched pairs test, p > 0.05. Abbreviations: NS not significant. (DOCX 526 kb) [file 13287_2016_465_MOESM4_ESM.docx]

**Additional file 4**

**Figure S3:** Stability of endogenous reference genes (TBP and YWHAZ) under differentiation conditions.

A) D)

B) E)

C) F)
