# Supplementary material for: Generation of mesenchymal stromal cells from cord blood: evaluation of in vitro quality parameters prior to clinical use
Source: Stem Cell Res Ther. 2017 Jan 24;8:14. doi: 10.1186/s13287-016-0465-2 (PMC5260040; doi:10.1186/s13287-016-0465-2)
Supplement: Additional file 5: Table S2. — List of antibodies used for immunophenotypic analysis. (DOCX 14 kb) [file 13287_2016_465_MOESM5_ESM.docx]

**Additional file 5**

**Table S2:** List of antibodies used for immunophenotypic analysis.

| Anti-human mAb | Clone | Conjugation | Supplier |
| --- | --- | --- | --- |
| CD90 | Clone F15-42-1-5 | FITC | Beckman Coulter |
| CD105 | Clone 1G2 | PE | Beckman Coulter |
| CD73 | Clone AD2 | Pe-Cy7 | Becton Dickinson |
| CD13 | L138 | Pe-Cy7 | Becton Dickinson |
| CD34 | 8G12 | PE | Becton Dickinson |
| CD31 | Clone 5.6E | FITC | Beckman Coulter |
| CD45 | Clone J.33 | ECD | Beckman Coulter |
| CD44 | Clone J.173 | FITC | Beckman Coulter |
| CD146 | Clone P1H12 | Pe-Cy7 | Becton Dickinson |
| CD271 | ME20.4-1.H4 | PE | Miltenyi Biotec |
| PDGFRB | Clone 18°2 | PE | Biolegend |
| HLA-ABC | Clone W6/32 | FITC | Biolegend |
| HLA-DR | Clone IMMU 357 | APC | Beckman Coulter |
| NG2 | 7.1 | PE | Beckman Coulter |
| CD54 | Clone HA58 | APC | Becton Dickinson |
| CD106 | STA | APC | Biolegend |
