# Supplementary material for: Generation of mesenchymal stromal cells from cord blood: evaluation of in vitro quality parameters prior to clinical use
Source: Stem Cell Res Ther. 2017 Jan 24;8:14. doi: 10.1186/s13287-016-0465-2 (PMC5260040; doi:10.1186/s13287-016-0465-2)
Supplement: Additional file 6: Table S3. — Five-color mAb combinations used for immunophenotypic analysis of inflammatory MSC priming. (DOCX 14 kb) [file 13287_2016_465_MOESM6_ESM.docx]

**Additional file 6**

**Table S3:** Five color mAb combinations used for immunophenotypic analysis of inflammatory MSC priming.

| Tube # | FITC | PE | ECD | APC | Pe-Cy7 |
| --- | --- | --- | --- | --- | --- |
| test tube #1 | CD90 | CD105 | CD45 | CD54 | CD73 |
| test tube #2 | CD90 | CD105 | CD45 | CD106 | CD73 |
| test tube #3 | HLA-ABC | CD105 | CD45 | HLA-DR | CD73 |
| FMO tube #1 | CD90 | CD105 | CD45 | FMO | CD73 |
| FMO tube #2 | HLA-ABC | CD105 | CD45 | FMO | CD73 |
| FMO tube #3 | FMO | CD105 | CD45 | HLA-DR | CD73 |
