# Supplementary figures and images for: Generation of mesenchymal stromal cells from cord blood: evaluation of in vitro quality parameters prior to clinical use
Source: Stem Cell Res Ther. 2017 Jan 24;8:14. doi: 10.1186/s13287-016-0465-2 (PMC5260040; doi:10.1186/s13287-016-0465-2)

**Additional file 7**

**Figure S4**

**A) B)**

**
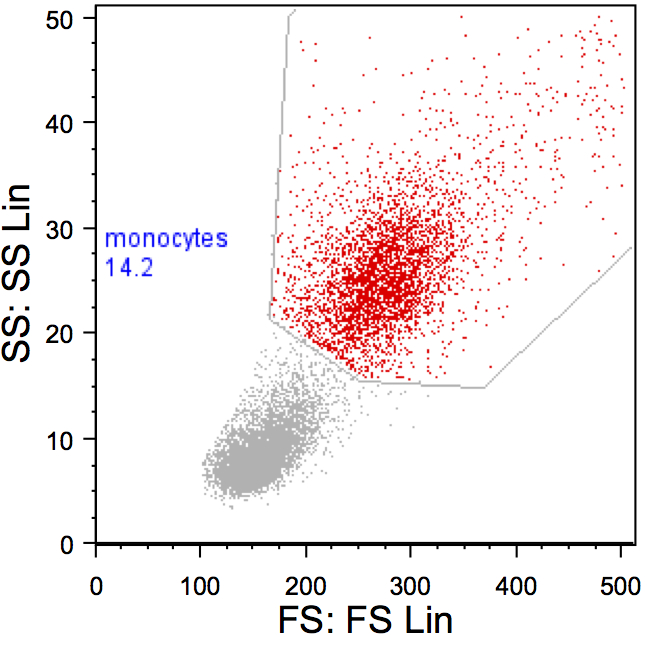

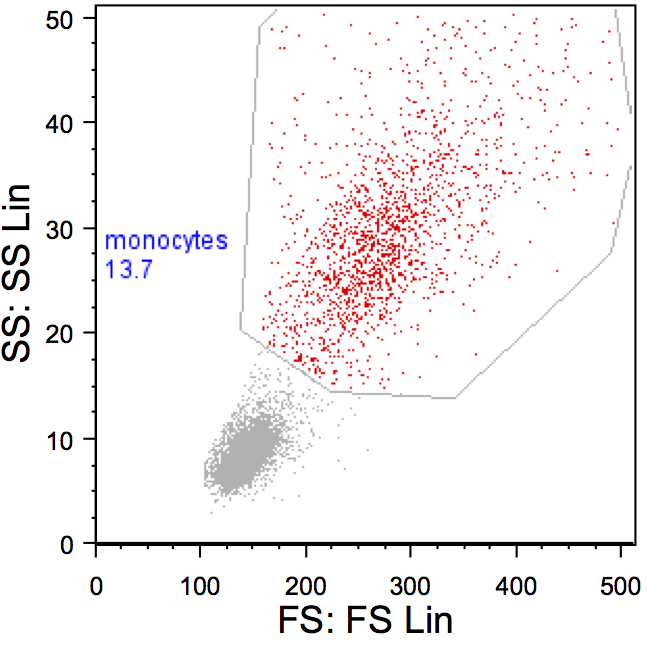
**

**C)**

Supplement: Additional file 7: Figure S4. — Immunomodulation assay. Flow cytometry analysis of thawed PBMC before (A) and after (B) overnight resting. The percentage of monocytes of one representative sample is shown. (C) Proliferation ratio between the percentage of CD45+ proliferation at primed and resting conditions, at the different MSC passages. Results from two independent experiments against two different PBMC lots are shown. (DOCX 637 kb) [file 13287_2016_465_MOESM7_ESM.docx]

**Additional file 8**

**Figure S5**

**
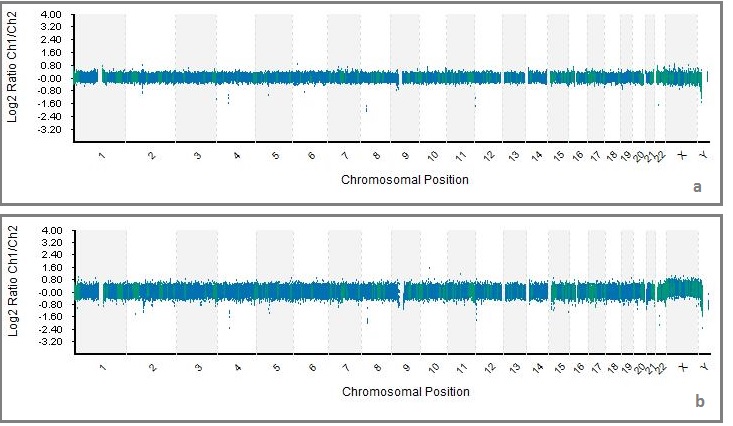
**

Supplement: Additional file 8: Figure S5. — Representative array-CGH profiles of the whole genome of one representative LL-CBMSC sample at P5 (lower panel) and P11 (upper panel) (DOCX 107 kb) [file 13287_2016_465_MOESM8_ESM.docx]

**Additional file 9**

**Figure S6:** SL-CBMSC multilineage differentiation

A)


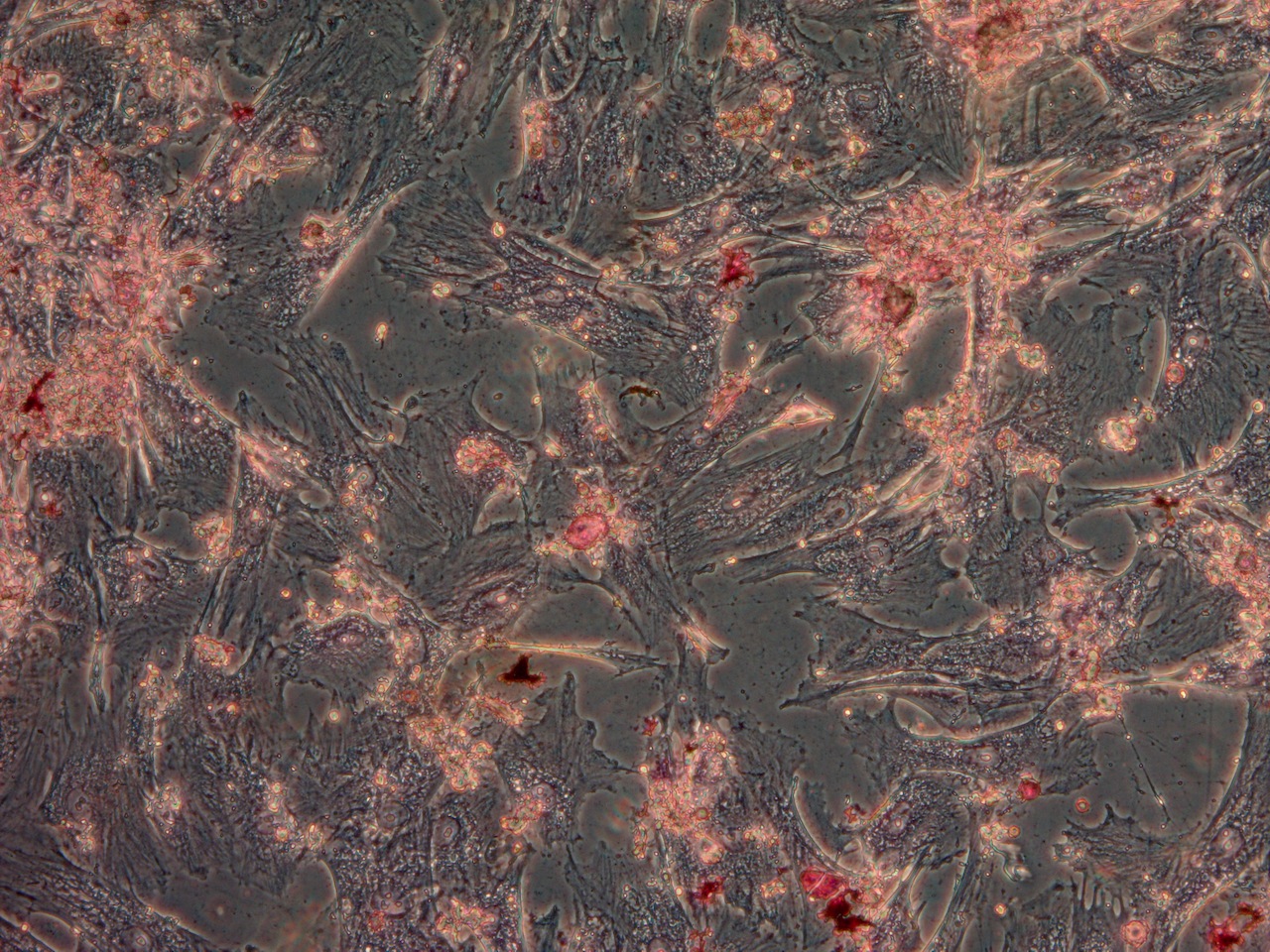


C)


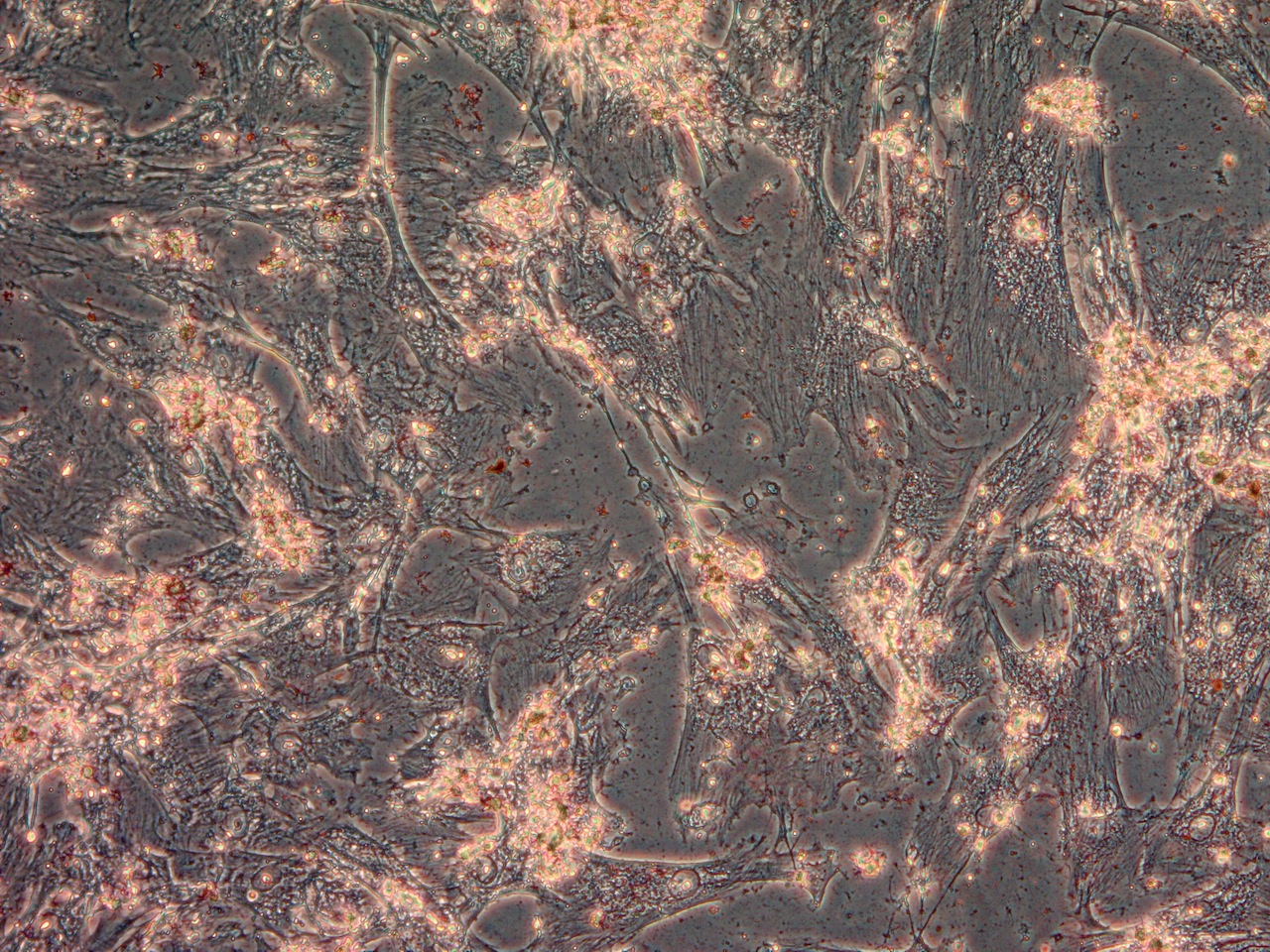


B)


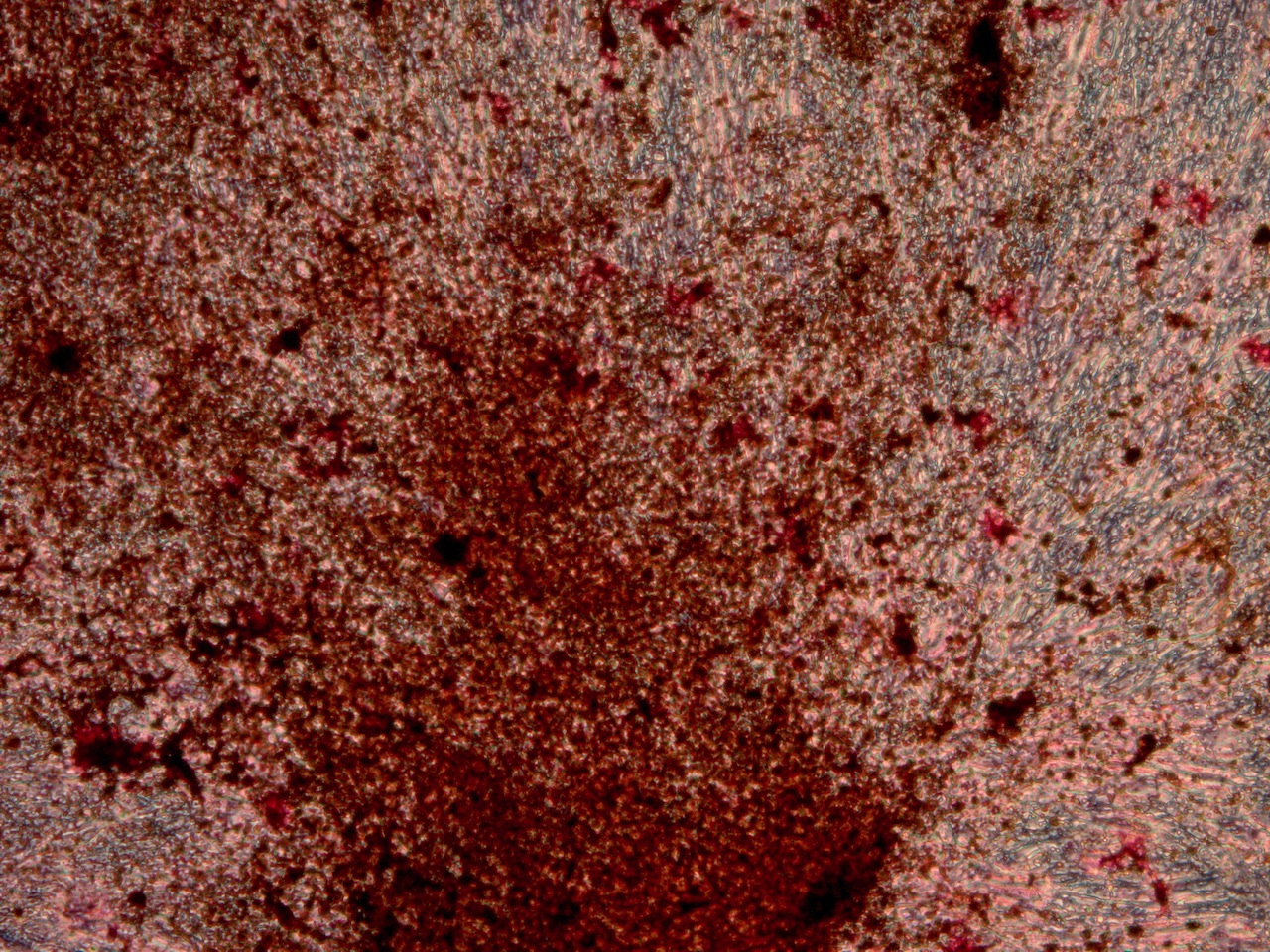


D)


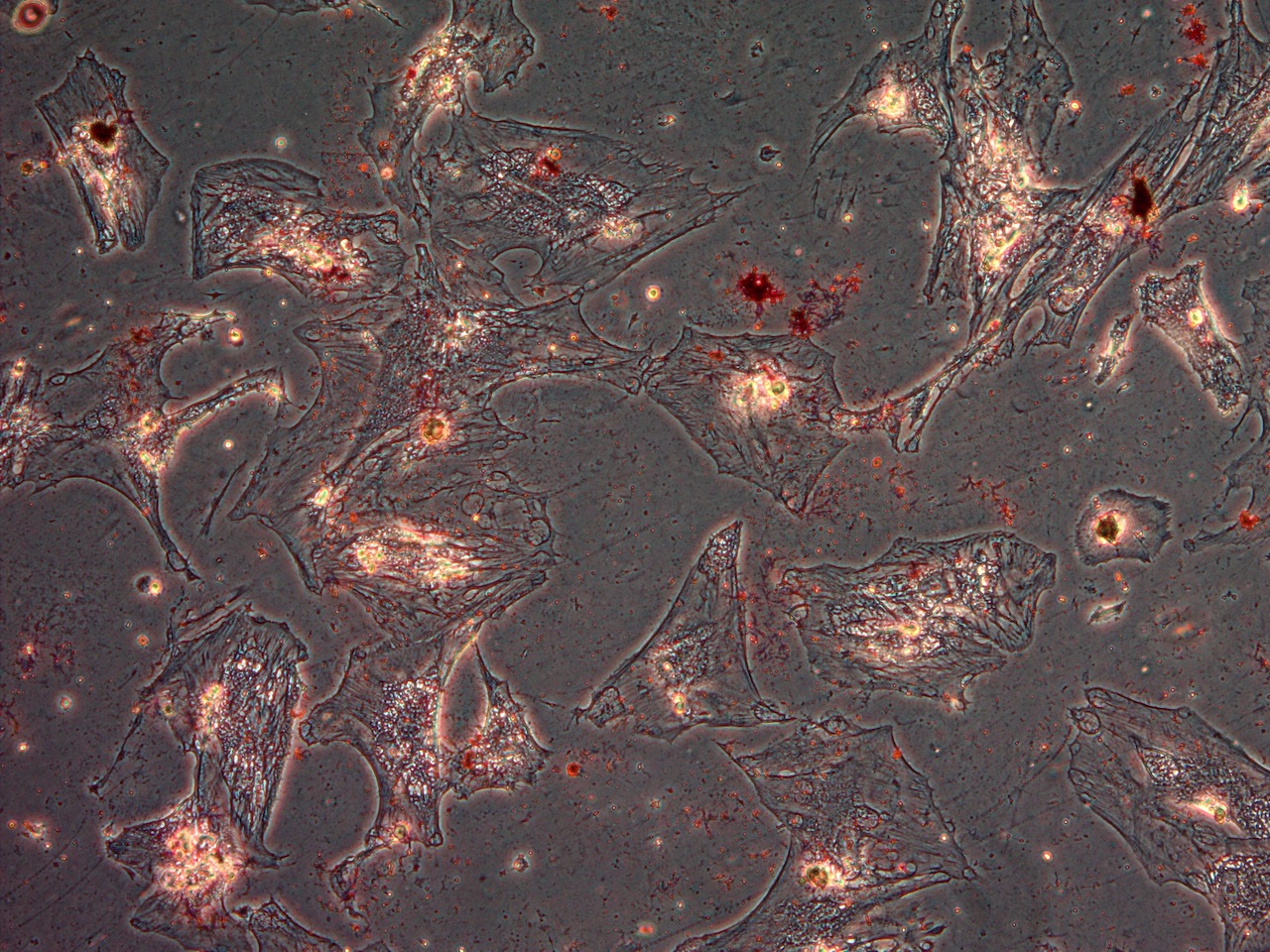

Supplement: Additional file 9: Figure S6. — SL-CBMSC multilineage differentiation. Panels display cells which have been induced to differentiate in vitro toward osteogenic (A-B) and adipogenic (C-D) lineages. Osteogenic and adipogenic differentiation were assessed after 21 days of induction using von Kossa and Oil Red O staining, respectively; ×10 magnification. For each staining, undifferentiated controls are also displayed on the left (panels A-C). One representative sample is shown. (DOCX 2.42 mb) [file 13287_2016_465_MOESM9_ESM.docx]
