# Supplementary material for: Generation of mesenchymal stromal cells from cord blood: evaluation of in vitro quality parameters prior to clinical use
Source: Stem Cell Res Ther. 2017 Jan 24;8:14. doi: 10.1186/s13287-016-0465-2 (PMC5260040; doi:10.1186/s13287-016-0465-2)
Supplement: Additional file 10: Figure S7. — SL-CBMSC immunophenotypic analysis. Characterization of SL-MSC (n = 6) by flow cytometry using a panel of 14 cell surface markers. Boxes extend from 25th percentile to the 75th percentile, the middle line represents median value and the whiskers extend from minimum to maximum values. Data are displayed as rMFI on the unstained control. (DOCX 104 kb) [file 13287_2016_465_MOESM10_ESM.docx]

**Additional file 10**

**Figure S7:** SL-CBMSC immunophenotypic analysis.
